# Probing myelin and axon abnormalities separately in psychiatric disorders using MRI techniques

**DOI:** 10.3389/fnint.2013.00024

**Published:** 2013-04-11

**Authors:** Fei Du, Dost Öngür

**Affiliations:** ^1^McLean HospitalBelmont, MA, USA; ^2^Department of Psychiatry, Harvard Medical SchoolBoston, MA, USA

**Keywords:** MTR, DTS, myelin, axon, g-ratio

## Abstract

In this manuscript we present novel MRI approaches to dissecting axon vs. myelin abnormalities in psychiatric disorders. Existing DTI approaches are not able to provide specific information on these subcellular elements but novel approaches are beginning to do so. We review two approaches (magnetization transfer ratio—MTR; and diffusion tensor spectroscopy—DTS) and the theoretical framework for interpreting data derived from these approaches. Work is ongoing to collect data that will answer some relevant questions using these techniques in schizophrenia and related conditions.

## Introduction

Mental illnesses are common, debilitating, and at times fatal (Kessler et al., 2005). Yet we know little about the pathophysiology of many of these conditions and existing treatments are partially effective and cause many side effects. Therefore, there is a great need for better insights into the neurobiology of psychiatric conditions and, as a corollary, for new treatment targets. Many lines of evidence suggest that white matter (WM) abnormalities are associated with psychiatric conditions. In fact, WM abnormalities are common in bipolar disorder (Heng et al., 2010), major depressive disorder (Arnone et al., 2012), and even attention deficit and hyperactivity disorder (van Ewijk et al., 2012). But the most pronounced and widespread abnormalities have been reported in schizophrenia. Therefore, we will discuss schizophrenia as a paradigmatic psychiatric disorder. In this review, we will discuss some of the evidence for WM abnormalities in schizophrenia, highlight what it can and cannot tell us about the biology and discuss emerging alternative approaches to the problem.

Schizophrenia is typically diagnosed at a young age, is life-long, and is among the leading causes of disability among people aged 15–35 (Global Burden of Disease, 2006). The cost of the illness is high and the suffering of patients with SZ and that of their families is great, as poor medication compliance, high rates of substance use disorder comorbidity, and suicide rates near 10% take their toll (Kessler et al., 2005). Despite its significance, relatively little is known about the pathophysiology of schizophrenia. Several lines of evidence suggest that integration of activity across brain regions is as important as processing within any one brain region. These include deficits in integration of activity in large-scale neuronal networks (Garrity et al., 2007; Williamson, 2007; Whitfield-Gabrieli et al., 2009), abnormalities in WM integrity (Kubicki et al., 2007; Camchong et al., 2009), and in expression of myelin- and oligodendrocyte-related genes (Tkachev et al., 2003). WM abnormalities are critical to conceptualization of SZ as a dysconnection (abnormal connection) syndrome (Paus et al., 2008; Stephan et al., 2009).

## MRI-based probes of white matter integrity

Although several MR parameters reflect tissue properties, the approach that is most widely used is diffusion MRI. As described elsewhere in this issue, water molecular diffusion, referring to the random translational (Brownian) motion of molecules, can be examined *in vivo* using diffusion MRI. The MRI signal decay when diffusion gradients are applied reflects the displacement distribution of water molecules. Because diffusion of water molecules is restricted by tissue components such as cell membranes or macromolecules, diffusion MRI provides unique information about the internal structure of brain tissue. In such experiments, a diffusion tensor is calculated and this consists of the three eigenvectors of diffusion arbitrarily labeled λ_1_, λ_2_, and λ_3_ from largest to smallest. Diffusion MRI has already been widely applied in the diagnosis and treatment of numerous brain disorders, most importantly in ischemic stroke where a fall in apparent diffusion coefficient [ADC; a.k.a. mean diffusivity = (λ_1_ + λ_2_ + λ_3_)/3] of water molecules is seen within hours of the ischemic event.

**Figure 1:**
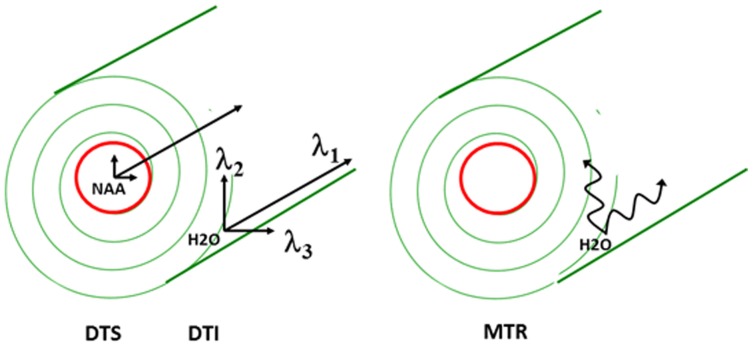
**Schematic of proposed white matter measures**.

In the WM, water molecular diffusion takes place along the fiber orientation direction [λ_//_ or axial diffusivity (AD) = λ_1_] to a much greater extent than perpendicular to it [λ_⊥_ or radial diffusivity (RD) = (λ_2_ + λ_3_)/2] (see Figure 1 for visual depiction of λ_1_, λ_2_, and λ_3_). This process can be measured using diffusion tensor imaging (DTI) where fractional anisotropy (FA—derived from λ_1_, λ_2_, and λ_3_) reflects coherence of diffusion. DTI has been used to demonstrate WM abnormalities in a variety of diseases including multiple sclerosis and schizophrenia. FA reductions are commonly interpreted as reflecting loss of “white matter integrity” but the exact nature of this loss cannot be determined using DTI because the abnormality could arise from intra- or extracellular water. In addition, there is exchange between the intra- and extracellular water compartments, making it impossible to deduce the biological source of any abnormalities. Therefore, reduced FA likely reflects different processes in different disorders (such as demyelination, fiber crossing, axonal swelling, or atrophy) (Alexander et al., 2007).

Despite these limitations, DTI studies have provided strong evidence that WM integrity has great functional significance. For example, variation in DTI measures in healthy populations has been associated with cognitive processing speed (Turken et al., 2008). In schizophrenia, FA reductions have been associated with specific clinical presentations such as passivity phenomena (Sim et al., 2009), auditory hallucinations (Shergill et al., 2007), or positive symptoms more generally (Fujiwara et al., 2007), cognitive functioning including working memory (Kubicki et al., 2003; Karlsgodt et al., 2008), episodic memory (Nestor et al., 2004, 2008), executive function (Nestor et al., 2004, 2008; Rusch et al., 2007; Takei et al., 2009), verbal learning (Takei et al., 2008), and visuomotor performance (Perez-Iglesias et al., 2010), and with fMRI connectivity measures (Schlosser et al., 2007; Spoletini et al., 2009). Some similar findings have been reported in prodromal individuals as well (Koutsouleris et al., 2010). This literature suggests that WM integrity is highly relevant to specific domains of brain function and dysfunction. In its current state, however, the literature is weakened by the lack of a clear link between DTI and WM biology (Kubicki et al., 2007) as evidenced by the absence of a deeper biological understanding of WM abnormalities in schizophrenia and of novel treatment targets.

## Novel approaches

The limitations of DTI as currently implemented in most centers and for studying psychiatric conditions are widely acknowledged. There are multiple approaches for extracting additional information from the diffusion signal in order to generate novel biological insights for psychiatric research. In this review, we provide a selective review of a two-pronged approach that focuses on axon and myelin-related abnormalities separately. An extensive review of all possibilities is far beyond the scope of this review. Instead, we will primarily focus on the directions our research group is taking and introduce concepts that are useful for understanding other approaches and briefly describe some alternative approaches at the end of the paper.

**Figure 2:**
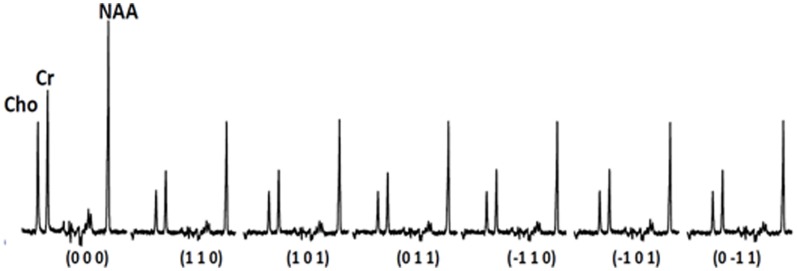
**Sample spectra acquired during the diffusion experiment**.

**Figure 3:**
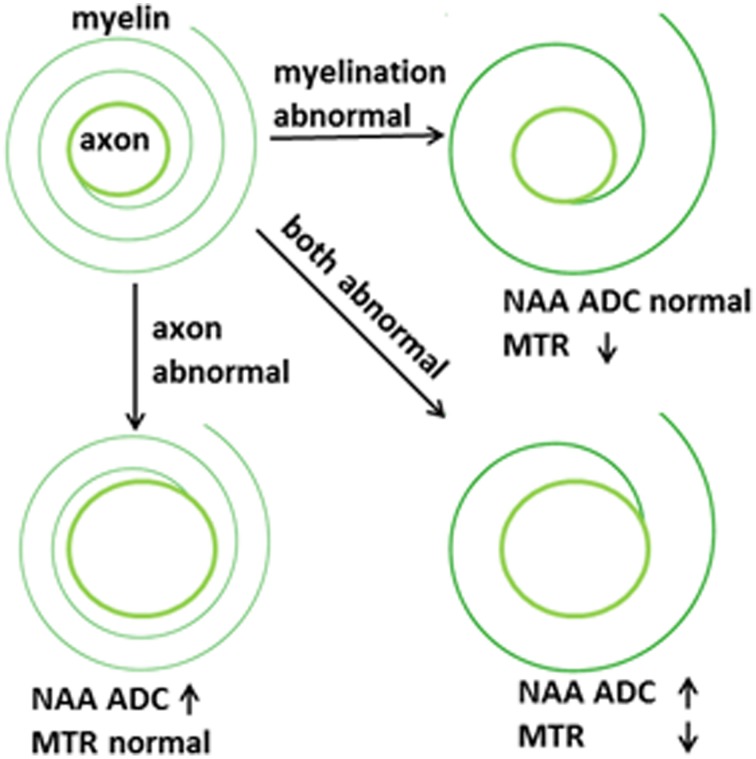
**Proposed abnormalities in schizophrenia**.

The two-pronged approach utilizes two recently developed MR-based approaches to probe specific WM abnormalities: diffusion tensor spectroscopy (DTS), and magnetization transfer ratio (MTR). DTS is a diffusion MRI technique related to DTI which measures the diffusion of intracellular metabolites such as N-acetylaspartate (NAA) (see Figure 2). Because NAA is located exclusively in neurons and almost exclusively in the cytosol (as opposed to within organelles) (Tsai and Coyle, 1995), NAA diffusion provides information about neuronal microstructure. The ADC reflects the scalar distance traveled by a molecule in unit time, and it is the easiest measure to interpret. In DTI studies in schizophrenia, for example, water ADC elevations have been reported accompanying the widespread water FA reductions discussed above (DeLisi et al., 2006; Andreone et al., 2007; Nenadic et al., 2011). DTS measures have three very useful properties: first, although molecular diffusion can reflect either physical hindrance by membranes or cytosol “viscosity,” data collection parameters can be modified to ensure that we measure primarily the former (e.g., by increasing diffusion times and *b*-values). Second, diffusion measures are independent of metabolite concentration. Therefore, any metabolite concentration abnormalities do not confound measures of metabolite diffusion. Third, ADC is sensitive to axonal geometry and less to the macroscopic curvature of WM tracts. If a voxel is placed at an angle to fiber direction or if fibers zigzag instead of traveling straight, ADC will vary less than FA or RD. This is because ADC is an average of the three λ's in the diffusion experiment and when fibers zigzag λ_1_ may go down but λ_2_ and λ_3_ rise, leaving their average approximately constant. In large voxel studies *in vivo* macroscopic curvature can often be a factor. Therefore, even though RD is intuitively more appropriate as an axon diameter measure, we focus on ADC. DTS approaches have been implemented and validated in previous work in a variety of contexts, including as probes of cellular diffusion (Ackerman and Neil, 2010) and for use in studies of axon diameter in the healthy WM (Upadhyay et al., 2008). The NAA DTS signal is informative: demyelination with preserved axon geometry would lead to no NAA ADC changes because NAA diffusion within axons would be unaffected. By contrast, axon diameter increase with preserved myelination would cause NAA ADC change (see Figure 3). It is important to note that the arguments presented above are relative, not absolute. NAA ADC can be impacted by multiple axon geometry factors such as macroscopic curvature and not just by axon diameter. While we propose that NAA ADC is useful in probing axonal geometry, it is only a partial index of axon diameter specifically. This is particularly true in brain regions such as the prefrontal cortex where there axons are not tightly packed.

MTR is another MRI modality that has garnered recent attention. This approach relies on the exchange of magnetization between water molecules in different physical environments. In biological tissue, water molecules form a thin film around macromolecules, including myelin lipids. The “bound” water molecules in this film exchange protons with the “free” water molecules in cytosol and this exchange can be measured using a magnetization transfer paradigm where signal is saturated in one component (e.g., bound water) and the loss of saturation in the other component is measured (e.g., free water). The larger the amount of myelin complement in WM, the larger the proton exchange between bound and free water, and the higher the loss of signal from free water. This loss of signal is quantified as MTR. MTR imaging is robust enough to be it is used in clinical settings to improve contrast in WM anatomical imaging, especially in multiple sclerosis. MTR is reported to be reduced in schizophrenia, suggesting reduced myelin complement in this condition (Kubicki et al., 2005) although a recent study was discrepant (Mandl et al., 2008). The relationships between water DTI and NAA DTS signal and longitudinal vs. radial diffusion as well as between MTR and WM microstructure are summarized in the adjacent Figure 1.

## Axon-myelin balance

Total WM volume is determined by the number of axons, their diameters, and the thickness of the myelin sheath around them. Although these parameters are inter-related, they are can vary partly independently. The importance of dissecting the processes that contribute to WM changes was highlighted recently in the context of normal human development. Increases in WM volume during adolescence have been traditionally interpreted as reflecting myelination but recent work using MTR showed that myelin content does not rise measurably in boys despite the fact that their WM volume increase is more pronounced than that of girls (Paus et al., 2008; Perrin et al., 2008, 2009; Paus, 2010). This pattern suggests that WM changes in male adolescence may be driven by increases in axonal diameter and number as opposed to an increase in the myelin complement.

The interplay between myelination and axon diameter is complex. Myelination speeds up conduction of action potentials. Larger axon diameters do the same, but the gain in conduction speed with growing axon diameters is less than that obtained from added myelination. Therefore, myelinated axons tend to be of small diameter to allow physical space for extra myelin. There appears to be a “sweet spot” for the ratio between axon diameter and fiber diameter (axon diameter + myelin sheath thickness) which maximizes conduction speeds. This is called the g-ratio and is calculated at around 0.6 for the human brain (Kandel et al., 1991; Chomiak and Hu, 2009; Paus and Toro, 2009). It is known that the g-ratio changes during brain development based on axonal electrical properties (Paus and Toro, 2009). In the extreme case, organisms with no myelin in the nervous system (such as the squid) have the largest axons, up to 1 mm in diameter (Chomiak and Hu, 2009; Paus and Toro, 2009). In pathological conditions where myelination is partial or degraded, axons can enlarge in diameter to compensate for reduced action potential speed (Nave, 2010).

## The challenge

Although DTI has been a very useful tool for psychiatric research, the ability to measure axon vs. myelin-related abnormalities separately in the WM is crucial for additional progress. DTI and MTR have been applied in schizophrenia, but there is no currently available measure that is axon-specific. A tool that provides axon specific along with myelin-related information would be valuable in identifying biologically meaningful abnormalities in this condition. MTR and DTS now provide this ability. In schizophrenia, there is strong evidence for myelination abnormalities (Hakak et al., 2001; Flynn et al., 2003; McCullumsmith et al., 2007; Uranova et al., 2007) as well as suggestions of a mechanistic relationship between developmental myelination abnormalities and schizophrenia (Budel et al., 2008). In addition, reductions in NAA levels have been reported in the WM in schizophrenia, suggesting a reduction in axonal packing density (Lim et al., 1998). Axonal diameter abnormalities have not been reported but until now these were only possible to measure in difficult *postmortem* electron microscopy studies. Axonal health and myelination are interrelated and abnormalities in one affect the other (Nave, 2010). Based on this literature we expect that application of DTS and MTR measures simultaneously will detect abnormalities in schizophrenia and in other psychiatric conditions. In this context, MTR functions as a marker of myelination and the NAA ADC as a marker of axonal geometry.

As reviewed above, correlation with cognitive and clinical outcomes have been used extensively to establish the pathophysiological significance of DTI abnormalities in schizophrenia. Armed with myelin- and axon-specific measures of WM integrity, it also becomes possible to explore the significance of each in similar fashion. For example, one would expect that cognitive tasks dependent on prefrontal circuitry may correlate with MTR and DTS measures obtained in the WM underlying the PFC. Another complementary approach to provide additional face validity for the combined MTR/DTS measures is the use of DTI to document abnormalities in water diffusion. The MTS/DTS approach can be used in conjunction with DTI (in fact, the water resonance measured during the DTS experiment is analogous to DTI data).

Taken together, the MTR/DTS approach would improve upon the existing paradigm of WM abnormalities in schizophrenia and other psychiatric conditions in two ways. First, it allows us to dissect the oft-repeated phrase “abnormal WM integrity” into component parts and examine how different changes in WM integrity can have different consequences. In fact, some WM alterations may be salutary ones as a compensation for upstream abnormalities. In this regard, the complex picture concerning WM changes during male vs. female adolescence discussed above provides a glimpse into how this proposal may challenge current paradigms of WM abnormality in psychiatry. Second, it offers concrete and measureable predictions about the impact of WM abnormalities on signal conduction in schizophrenia. I.e., “disrupted WM integrity” cannot correlate with conduction speed but axon geometry and myelin sheath thickness can. Although many have noted that WM abnormalities in schizophrenia must have functional consequences (Kubicki et al., 2007), the link between DTI measures and brain function remains abstract (Whitford et al., 2012).

Despite great interest in the role WM abnormalities play in cognitive function in psychiatric conditions, we do not yet have WM treatment targets. This is partly because existing measures of WM abnormalities are underdetermined, i.e., several mechanisms could lead to the same result. Therefore, we do not know which biological mechanisms are responsible for the WM alterations seen in schizophrenia. We propose that the MTR/DTS measure will offer meaningful biomarkers for this critical process.

There is a lengthy and productive history of studying *postmortem* tissue to probe WM abnormalities in psychiatry (e.g., Benes et al., 1987; Akbarian et al., 1993; Selemon and Goldman-Rakic, 1999), but *in vivo* neuroimaging tools have not been applied systematically to this problem except through the use of DTI. In addition, inherent problems with fixation often limit the application of electron microscopy to many *postmortem* human brain samples. Abnormalities in axons and in myelin have been reported, but we have had no ability to detect or monitor these *in vivo*. Although the application of MRI to histology has been discussed previously as “magnetic resonance microscopy” (Mori and Zhang, 2006; Mori et al., 2006), these ideas have not yet been expanded to human psychiatric disorders. Our vision is to expand MTR/DTS and related approaches into a “toolbox” measuring several aspects of the brain microenvironment in clinically acceptable scan times. To be maximally useful, this toolbox would collect regionally-specific data from voxels within gray matter (GM) and WM, and would include T1 and T2 relaxation times (Ongur et al., 2010) and perhaps other measures as well as MTR/DTS. Although this is a challenge with current technology, increasing field strengths and improving technical approaches are increasingly making it possible to use neuroimaging to probe parameters previously accessible only to *postmortem* research.

## Magnetization transfer ratio (MTR) spectroscopy

**Figure 4:**
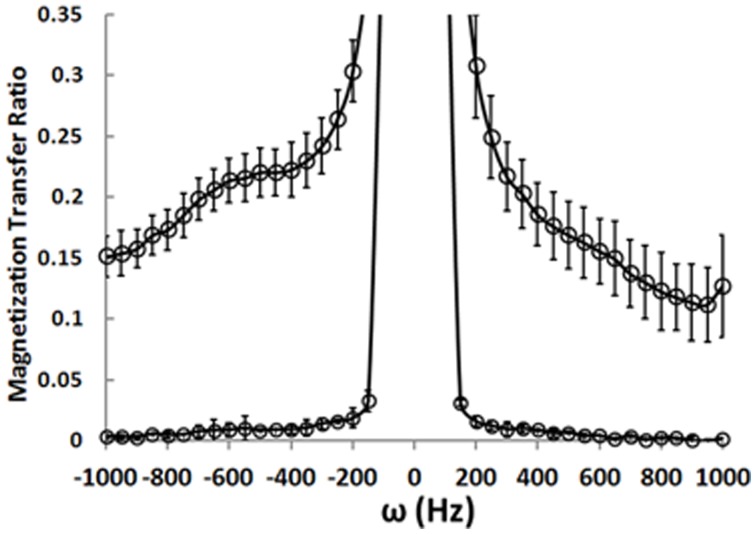
**Magnetization transfer ratio data**.

We have implemented the MTR sequence on the 4T Varian scanner at McLean Hospital and collected MTR data from a phantom preparation containing an aqueous NaCl-solution. The MTR experiment relies on measuring the total magnitude water signal in the presence and absence of a BISTRO saturation pulse. The water signal magnitude is maximal without the saturation pulse. The pulse causes saturation of signal coming from “bound” water molecules. Because there is transfer between “bound” and “free” water molecules, the saturation pulse measurably attenuates the signal coming from “free” water molecules. Figure 4 shows the MTR measured after a saturation pulse is applied at resonances near the main water resonance (normalized to a non-pulse intensity of 1.0). The lower curve represents measurements made in the aqueous solution phantom where there are no “bound” water molecules. When the saturation pulse is applied at most frequencies the MTR is zero, but it rises when the pulse is positioned right on the water frequency (i.e., it saturates all water molecule resonances). The data points from a healthy control collected from a 1 × 3 × 3 cm (9 cc) voxel in the WM underlying the right PFC show that, as expected, the saturation pulse causes a rise in MTR even when it is off-resonance in the human brain. This is because there are “bound” water molecules *in vivo* interacting with lipids and proteins and these are affected by the off-resonance saturation pulse. Their chemical exchange with the “free” water molecules causes a loss of water signal intensity. The MTR can be calculated based on water signal intensity acquired in presence (M_s_) and absence (M_c_) of the BISTRO saturation pulse [MTR = (Mc-Ms)/Mc].

Compared to DTI, MTR is equally quick to obtain whole brain data. But the two techniques measure different processes, each with its own limitations. Therefore, the two techniques are best considered complementary.

## DTS

We have also implemented DTS measurements of water and metabolites on the same 4T Varian scanner at McLean from the same 9 cc right PFC WM voxel in the human brain. DTS measurements in a phantom preparation demonstrated isotropic diffusion and these measures were used to calibrate diffusion gradient strength (*b*-value). The voxel location is identical to that in the MTR experiments. The seven spectra obtained during a DTS experiment are shown in Figure 2 with the NAA peak highlighted in select spectra. Note that we show water-suppressed spectra here for simplicity, although we also collected water unsuppressed spectra which allow us to calculate water ADC and FA. From these spectra are calculated the three diffusion eigenvalues and subsequently the ADC, RD, AD, and FA. The units for the diffusion data are mm^2^/s × 10^−3^. The water FA and ADC and the NAA FA and ADC values we calculate for a healthy control are similar to those in other DTI and DTS studies (e.g., Upadhyay et al., 2008; Camchong et al., 2009). Specifically, NAA is a larger molecule and diffuses more slowly than water so it has a lower ADC. Furthermore, two observations confirm that NAA has the diffusion properties of an intracellular metabolite while water does not: NAA FA is much higher than water FA; and the NAA AD/RD ratio is higher than the water AD/RD ratio. Being restricted within axons would increase FA and the AD/RD ratio, exactly as seen with NAA. The findings for creatine and choline (two other intracellular metabolites which are quantified with DTS) were similar to those for NAA. The metabolite diffusion parameters measured in a sample of 10 healthy individuals are listed in Table 1 below. These studies showcase our ability to implement a cutting-edge MRI sequence at 4T and to collect data on WM microstructure.

**Table 1:** **Diffusion data in the healthy human white matter**.

	**RD**	**AD**	**ADC**	**FA**
NAA	0.16 ± 0.04	0.41 ± 0.09	0.24 ± 0.04	0.55 ± 0.15
Cr	0.15 ± 0.04	0.40 ± 0.08	0.23 ± 0.03	0.58 ± 0.18
Cho	0.15 ± 0.04	0.43 ± 0.10	0.24 ± 0.05	0.60 ± 0.10

Compared to DTI, DTS collects data more slowly and from a single large voxel (as opposed to small voxels with whole brain coverage in DTI). These shortcomings are balanced by the different information offered by DTS—the diffusion properties of NAA are dependent on axon geometry and this information is complementary to the water diffusion information garnered using DTI.

## Alternative approaches

Some shortcomings of the single-voxel MTR/DTS approach would be remedied if we could collect data from the entire brain as is done in DTI studies. This is possible for MTR but not for DTS currently because metabolites such as NAA are present in the brain at about 1:5000 the concentration of water (approximately 10 mM vs. 50 M). This dramatically reduced signal forces us to collect data from a larger voxel and to carry out more repetitions for each data point to reach acceptable signal-to-noise ratios. Chemical shift imaging approaches have been described which can collect high quality MRS data from the entire brain (Posse et al., 2007; Maudsley et al., 2009) but these have not yet been combined with diffusion gradients. We plan to explore this combination but we recognize that this may not be possible given the SNR limitations.

Another concern with our DTS approach is that the NAA signal we measure in our experiments contains contributions from NAA and N-acetylaspartylglutamate (NAAG). NAAG is located both intra- and extracellularly (Coyle, 1997) and our DTS measures may be confounded by this contamination. NAAG is very similar to NAA in chemical structure so the two MRS signals are challenging to resolve using regular PRESS approaches. NAAG concentration in the PFC WM is 1.5 mM in healthy individuals (of which an unknown fraction is extracellular) (Pouwels and Frahm, 1997) whereas NAA concentrations are usually calculated at about 10 mM (Govindaraju et al., 2000). Therefore, we do not expect this to be a major factor in our work.

Finally MTR, although sensitive, is not a specific measure of myelin content. Concerns have been raised that MTR abnormalities can arise from acute inflammation, edema, and other processes that impact brain water content (Laule et al., 2007). This issue limits the utility of MTR in pathologies where gross abnormalities in brain water content are seen. There is no evidence for such gross abnormalities in psychiatric disorders and past applications of MTR in schizophrenia have been consistent with subtle myelination-related changes (Kubicki et al., 2005). Others have proposed a more specific measure of WM myelin content which takes advantage of the differential T2 relaxation properties of water trapped within myelin vs. free water (MacKay et al., 2006). This approach may be desirable because it would be more specific to myelin content, but it is more technically challenging to implement. The T2-based approach requires using ultrashort echo times (5 ms or shorter) which in turn require optimization of both hardware (amplifiers, transmit-receive switches) and software (e.g., STEAM sequences as opposed to our usual PRESS sequences).

There are also alternatives to the DTS approach, e.g., by optimizing the water diffusion experiment parameters for measuring axon diameter. Since the water signal is so much larger, such data could theoretically be collected in a shorter time than DTS. One such approach (termed AxCaliber) is in fact in early stages of development but thus far it has only been validated *in vitro* (Assaf et al., 2008). Unfortunately AxCaliber requires sequences to be repeated over very long scan times—and as a result is not available yet for clinical studies. Other approaches are also being developed (Zhang et al., 2011; Dyrby et al., 2012). Thus, we believe the single voxel DTS approach currently represents the state-of-the-art for examining changes in axon geometry.

## Conclusions

A better understanding of WM abnormalities would be critical for the development of better treatments for common psychiatric conditions. Although DTI has shown us that these conditions are characterized by reduced WM integrity, it cannot tell us whether the abnormality is in axons, myelin, or both. This information is relevant because signal transduction in the WM depends on the relative health of these two compartments. Recently developed techniques such as MTR and DTS allow us to dissect signal from these two compartments, which would in principle help us quantify their relative abnormalities in psychiatric conditions. Many challenges remain, foremost among them the limitation of DTS data collection to a single large voxel and the difficulty of interpreting DTS signal from such large voxels. But future clinical studies will give us more detailed information on WM abnormalities in schizophrenia and other conditions than we have previously had access to. In addition, the application of these methods can extend beyond psychiatric disorders to study of aging and gender comparisons which may have additional implications for cross sectional and longitudinal evaluations in psychiatric studies.

### Conflict of interest statement

The authors declare that the research was conducted in the absence of any commercial or financial relationships that could be construed as a potential conflict of interest.
